# Toward Current Matching in Tandem Dye-Sensitized Solar Cells

**DOI:** 10.3390/ma13132936

**Published:** 2020-06-30

**Authors:** Junfeng Wei, Zhipeng Shao, Bin Pan, Shuanghong Chen, Linhua Hu, Songyuan Dai

**Affiliations:** 1 Key Laboratory of Photovoltaic and Energy Conservation Materials, Hefei Institutes of Physical Science, Chinese Academy of Sciences, Hefei 230031, China; jfwei@outlook.com (J.W.); shaozp@mail.ustc.edu.cn (Z.S.); panbinsp@126.com (B.P.); lhhu@rntek.cas.cn (L.H.); 2 Beijing Key Laboratory of Novel Thin-Film Solar Cells, Beijing Key Laboratory of Energy Safety and Clean Utilization, North China Electric Power University, Beijing 102206, China

**Keywords:** dye-sensitized solar cells, pn type tandem, circuit model, photocathode, current matching

## Abstract

The tandem pn-type dye-sensitized solar cells (pn-DSCs) have received much attention in the field of photovoltaic technologies because of their great potential to overcome the Shockley-Queisser efficiency limitation that applies to single junction photovoltaic devices. However, factors governing the short-circuit current densities (*J*_sc_) of pn-DSC remain unclear. It is typically believed that *J*_sc_ of the pn-DSC is limited to the highest one that the two independent photoelectrodes can achieve. In this paper, however, we found that the available *J*_sc_ of pn-DSC is always determined by the larger *J*_sc_ that the photoanode can achieve but not by the smaller one in the photocathode. Such experimental findings were verified by a simplified series circuit model, which shows that a breakdown will occur on the photocathode when the photocurrent goes considerably beyond its threshold voltage, thus leading to an abrupt increase in *J*_sc_ of the circuit. The simulation results also suggest that a higher photoconversion efficiency of the pn-DSCs can be only achieved when an almost equivalent photocurrent is achieved for the two photoelectrodes.

## 1. Introduction

Dye-sensitized solar cells (DSCs) have attracted a great deal of attention during the last three decades due to their projected low cost and comparably facile assembly. A typical DSC is constructed by sandwiching a photocathode which is usually a dye-sensitized TiO_2_ and a counter electrode. Although lots of research and development of these systems have been carried out since the initial breakthrough in 1991, the same concept is still used [1]. To make DSCs more competitive in future utilization, the next big step is to improve efficiency up to 15–20% [2]. Therefore, an apparent and simple way to improve the efficiency of the DSC is to construct a tandem structure DSCs [3]. The theoretical upper limit for a typical dye-sensitized solar cell is around 30%. Tandem solar cells (t-SCs), compared with single junction solar cells, have higher theoretical efficiency limitations [4]. For two junction solar cells, the theoretical efficiency limitation is around 42% [5,6]. Recently, the most efficient solar cells are operated in tandem structure. The most efficient two junction devices have reached a *PCE* (Power Conversion Efficiency) over 30% [7,8]. However, compared with single junction solar cells, the fabrication of t-SCs is always more complicated, which may significantly limit their utilization. However, Pn-DSCs which combine a photoanode and a photocathode into a single device represent an exceptional case [9,10]. As two junction solar cells, pn-DSCs have a simple preparation process which is almost the same as the traditional single junction dye-sensitized solar cells (n-DSCs or p-DSCs) [11,12,13,14]. These advantages make them more competitive in future applications.

The first pn-DSC with an efficiency of 0.39% was reported in 2000 [6]. Since then, an increasing number of research articles have been published in this area [11,15,16,17,18,19]. Suzuki and co-workers reported a pn-DSC with a better-quality mesoporous NiO photocathode achieving a *PCE* of 0.66% with a *J*_sc_ of 3.62 mA/cm^2^ [20]. In 2009, Elizabeth A. Gibson et al. reported a pn-DSC with a *PCE* of 0.55% [21]. In 2010, A. Nattestad et al. reported a well optimized pn-DSCs with a record *PCE* of 1.91% [9]. In 2012, an Se based photocathode for pn-DSC was developed with a *PCE* of 0.98% [22]. Recently, J.H.Kim et al. improve efficiency through the blocking layer at the photocathode interface to 1.91% [23].

Current mismatch between the photocathode and the photoanode is one of the most critical issues limiting the performance of pn-DSCs, and thus may lead to poor *FF* (Fill Factor) or *PCE* of the pn-DSCs lower than that of the corresponding n-DSCs [19,24,25]. In this paper, we discuss the problems of current mismatch and summarize how to get a good current match in pn-DSCs. The *J*_sc_ of the pn-DSCs is considerably governed by the weakest photoelectrode. In our study, we found that the *J*_sc_ of the pn-DSCs is always determined by the photoanode, no matter which photoelectrode is weaker. We adopted a simple series circuit equation to model the *J-V* curve of pn-DSCs which corresponded n-DSC and p-DSC. Furthermore, this work studied the Pn-DSCs with the same photocathode and a different performance photoanode. The calculation results show good agreement with the experiment.

## 2. Experimental

**Figure 1:**
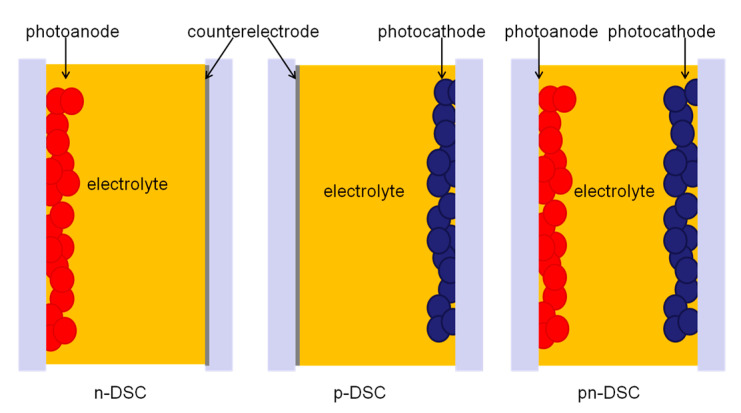
Structure of n-DSC, p-DSC and pn-DSC.

The structures of pn-DSC, n-DSC and p-DSC were illustrated in Figure 1. Pn-DSC was fabricated by sandwiching a typical photoanode with a polymer based photocathode. The n-DSC was fabricated by sandwiching a typical photoanode with a Pt counter electrode. A photoanode with a different photocurrent was prepared by changing the TiO_2_ thickness. The p-DSC was fabricated by sandwiching a polymer based photocathode with a Pt counter electrode. The typical photoanode was constructed as described elsewhere [26]. A transparent, mesoporous TiO_2_ film was prepared by sintering TiO_2_ paste-coated FTO (Fluorine-doped SnO_2_ transparent conductive glass) at 510 °C for 30 min. The mesoporous TiO_2_ films were then cooled to 80 °C and immersed in an ethanol solution of a ruthenium-complex (N719 dye) overnight, followed by rinsing in ethanol and drying. The polymer based photocathodes were fabricated as follows: mesoporous NiO film was fabricated on FTO by spin coating, followed by sintering at 510 °C for 30 min under atmospheric. Spin coating paste was produced by mixing a slurry of 10 g of NiO nanopowder (Sigma Aldrich, Saint Louis, MO, USA) in ethanol with 30 g of 10 wt% ethanolic ethyl cellulose (Sigma Aldrich, Saint Louis, MO, USA) solution, 35 g terpineol, and 75 g ethanol. Then spin-cast a blend chlorobenzene solution containing PCPDTBT (Poly[2,1,3-benzothiadiazole-4,7-diyl[4,4-bis(2-ethylhexyl)-4H-cyclopenta[2,1-b:3,4-b’]dithiophene-2,6-diyl]]) and PCBM ([6,6]-phenyl-C61-butyric acid methyl ester) (15 mg/mL, 35 mg/mL) on the mesoporous NiO film, followed by removing the PCBM by simply soaking this hybrid film in 3-methoxypropionitrile (MePN). Electrolytes used for all n-DSCs, p-DSCs and pn-DSCs were similar (0.6 M N-methyl-N-butylimidazolium iodide, 0.45 M N-Methyl benzimidazole, 0.1 M LiI and 0.1 M I_2_ in 3-Methoxypropionitrile).

The photovoltaic performances of the DSCs were measured using a Keithley 2420 3A source meter (Tektronix, Beaverton, OR, USA) controlled by Labview software under AM 1.5 solar simulator of 100 mW cm^−2^ (solar AAA simulator, oriel USA, calibrated with a standard crystalline silicon solar). The active area of the samples was 0.25 cm^2^ (with a black mask). Absorption spectra were obtained from a UV-vis spectrophotometer (U-3900H, Hitachi, Tokyo, Japan). 

## 3. Results and Discussion

Photocurrent matching is an essential prerequisite for the realization of highly efficient pn-DSCs. However, the reported photocurrent of photocathode ever reported is still far behind the well optimized photoanode, and thus can lead to the significant photocurrent mismatch in pn-DSCs.

**Figure 2:**
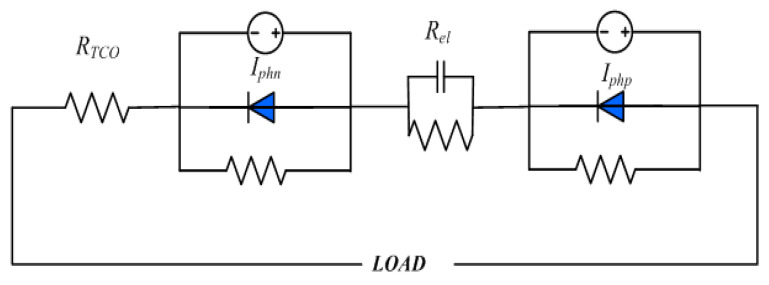
The simplified equivalence circuit of the pn-DSC.

Equivalence circuit analysis has been widely used in DSCs to study the electric mechanism [27,28]. For pn-DSCs, an equivalence circuit can be simplified as a series connection of a n-DSC and a p-DSC (as shown in Figure 2) [29]. Here we adopted a simplified series circuit model that has been used in series connection DSCs to calculate the *J-V* characters of pn-DSCs which corresponded n-DSC and p-DSC [30,31,32]. In this model, we used *J-V* characters of the corresponding n-DSC and p-DSC to replace the actual *J-V* characters of the photoelectrodes.

**Figure 3:**
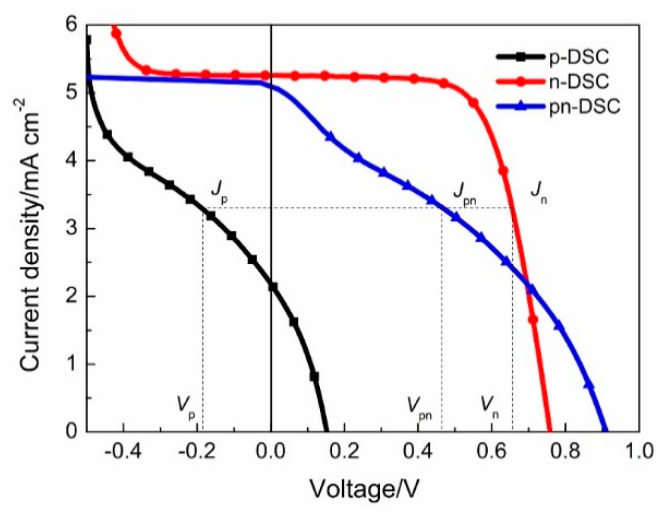
The simulated *J-V* curve of pn-DSCs from the corresponding n-DSC and p-DSC.

Because of the series connection of two photoelectrodes in pn-DSCs, the photoanode and the photocathode work in the same current, whereas the voltage is the sum of two photoelectrodes as shown in Figure 3. The voltage and current of the pn-DSCs can be expressed as the following Equations (1) and (2):

(1) $$V_{\mathrm{pn}}=V_{p}+V_{n}$$

(2) $$J_{pn}=J_{p}=J_{n}$$

**Figure 4:**
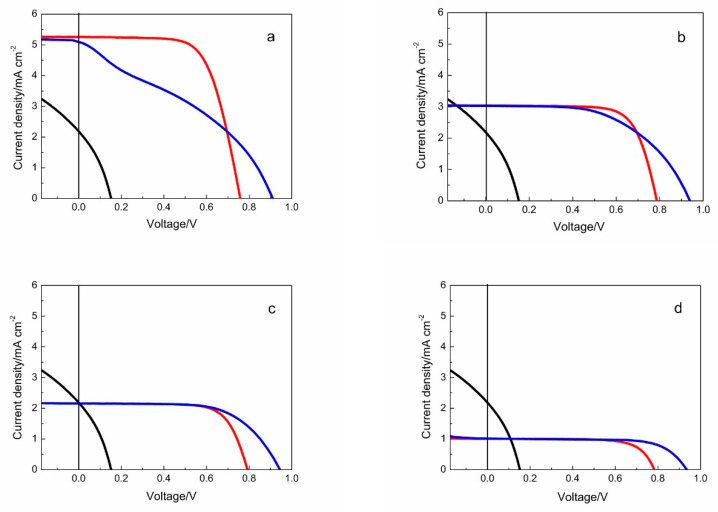
*J-V* curves of the calculated pn-DSC (blue line) as well as the corresponding n-DSC (red line) and p-DSC (black line). (**a**) Pn-DSC with a high performance photoanode and the photocathode; (**b**) Jsc of n-DSC in the range from Jsc to the threshold photocurrent density of the p-DSC; (**c**) Photocurrent of the two photocathode are well matched; (**d**) Jsc of the n-DSC lower than that of p-DSC.

The reported P-DSCs in the literature that are based on the I^−^/I_3_^−^ electrode and NiO exhibited a very low *V*_oc_ typically below 0.2 V and a *FF* between 30–40% [33]. Figure 4 shows the typical *J-V* characteristics of a p-DSC from our experimental samples. Details of *J-V* characteristics are shown in Table 1, the p-DSC exhibits a *PCE* of 0.12%, a *V*_oc_ of 0.15 V, a *J*_sc_ of 2.15 mA cm^−2^ and a *FF* of 33.5%. Under negative bias, photocurrent density increases with the applied negative bias. When the negative bias exceeds 0.4 V (the threshold voltage of p-DSCs), there is a breakdown of p-DSCs, which exhibits a sharp increase in photocurrent density.

Figure 4 shows the simulated *J*-*V* characters of pn-DSCs with the photocathode mentioned above and a changed photoanode (the different performance of photoanode are achieved by adjusting the TiO_2_ thickness): (a) Pn-DSC with a high performance photoanode and the photocathode. The corresponding *J*_sc_ of n-DSC is far larger than that of p-DSC, even larger than the threshold current density of p-DSC; (b) *J*_sc_ of n-DSC in the range from *J*_sc_ to the threshold photocurrent density of the p-DSC. (c) Photocurrent of the two photocathode are well matched; (d) *J*_sc_ of the n-DSC lower than that of p-DSC. All *J-V* curves of the n-DSCs and the p-DSC are achieved from our experiments.

Pn-DSCs and DSCs were illuminated through the photoanode side and the PSCs (Polymer Solar cells) through the photocathode side.

Figure 4a shows the *J-V* curve under the first condition. The corresponding n-DSC exhibit a *PCE* of 2.68% with a *V*_oc_ of 0.76 V, *J*_sc_ of 5.26 mA cm^−2^, which is larger than the threshold current density of p-DSC, and a *FF* of 67.2% (Photovoltaic parameters of pn-DSCs, as well as p-DSCs and n-DSCs, are shown in Table 1). The *J-V* curve of the pn-DSC shows an S slope character with a low *FF* of 35.2%. The threshold voltage of p-DSC with this polymer based photocathode is between 0.4–0.5 V, which is much lower than the *V*_oc_ of a typical n-DSC. Under the open circuit condition, the photocathode works under positive bias to get a constant current and thus leads to the *J*_sc_ of pn-DSCs which is similar with n-DSC. This is quite different from the natural consideration, and the photocurrent of pn-DSC is limited by the smaller one of the two photoelectrodes. Even though the open circuit voltage of the pn-DSC is a summation of the corresponding n-DSC and p-DSC, the voltage of the maximum power point (*V_max_*) has only a little increase (Table 1). In this condition, *PCE* of pn-DSCs (1.64%) is far behind the n-DSCs (2.68%). The previous pn-DSC did not achieve current matching, the fill factor is very low, and it is in the first state of our calculation [6,21].

Under the second condition, the *J*_sc_ of the corresponding n-DSC decreased to 3.04 mA cm^−2^ which is in the range from *J*_sc_ to the threshold photocurrent density of the p-DSC, as shown in Figure 4b. A normal *J-V* curve was obtained. The *FF* of pn-DSC increased to 54.5% as compared to the first condition. However, the *PCE* of pn-DSCs is still smaller than that of n-DSCs. The *J*_sc_ and the *V*_mav_ of the pn-DSCs are almost the same as that of n-DSCs.

Figure 4c shows the *J-V* curve of pn-DSC when the *J*_sc_ of the two single cells are well matched. The *J*_sc_ of the pn-DSCs are the same as the p-DSC and n-DSC. The *FF* of the pn-DSC reaches 64.0%, while still being lower than that of the n-DSC (72.3%) which is hampered by the low *FF* of the p-DSCs. Under this condition, both the *PCE* and the *V*_max_ slightly increase as compared with the corresponding n-DSCs (Table 1). Nattestad et al. obtained a fill factor of 74% by adjusting the current matching between the photocathode and the photoanode, resulting in a photoelectric conversion efficiency of 1.91% [9]. This is consistent with our simulation calculations.

*J*-*V* characters of pn-DSCs with the *J*_sc_ of the corresponding n-DSC are lower than that of the p-DSC as shown in Figure 4d. The n-DSC used here shows a *PCE* of 0.57% with a *V*_oc_ of 0.78 V and *J*_sc_ of 1.01 mA cm^−2^. The resulting pn-DSC exhibits a *PCE* of 0.67%, which is a considerable enhancement compared to the corresponding n-DSC. Furthermore, the *V*_max_ shows an apparent increase from 0.64 V to 0.73 V. The *FF* of the pn-DSC is closer than the n-DSC, which indicates that a higher *J*_sc_ of the photocathode could result in a better *FF*. But the PCE of pn-DSC under this condition is lower than that of photocurrent matching.

Pn-DSCs and DSCs were illuminated through the photoanode side and the PSCs through the photocathode side.

**Figure 5:**
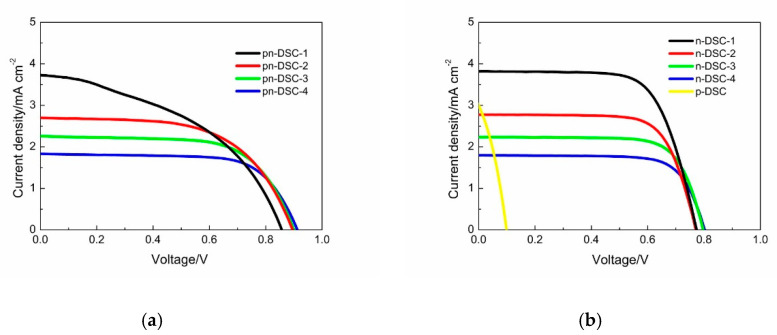
(**a**) *J-V* curve of the pn-DSC with different photoanode; (**b**) *J-V* curve of the corresponding n-DSC and p-DSC.

Figure 5a shows the experimental result of the pn-DSCs with the same photocathode and the changed photoanode. Sensitizers of the two photoelectrodes exhibited good complementary absorption, and thus the influence from absorption of the photoanode to the photocathode is small. The *J-V* characters of the corresponding n-DSCs and p-DSC with the same photoelectrode are shown in Figure 5b (pn-DSC and n-DSC are illuminated from the photoanode side and the p-DSC are illuminated from the photocathode side). The photovoltaic parameters of pn-DSCs, p-DSCs and n-DSCs are shown in Table 2. The results from our experiment show a good agreement with the calculated values. The *V*_oc_ of the pn-DSCs is the sum of the n-DSC and the p-DSC. *J*_sc_ of the pn-DSC are determined by the n-DSC, and the *FF* of pn-DSC increases when the *J*_sc_ of the n-DSC decrease. It is hard to determine the actual *J*-*V* character of the photocathode in pn-DSC. From the *FF* of the pn-DSC as compared to the calculation result, the pn-DSC with a *FF* of 65.3% (green line) could be considered close to a good current match, and the *PCE* of 1.33% is slightly larger than the 1.31% of the corresponding n-DSCs, also meeting the calculation result.

## 4. Conclusions

In conclusion, we discussed the problem of current mismatch in pn-DSCs which are constructed from a typical low performance photocathode and a high performance dye-sensitized TiO_2_ photoanode. By adopting a simplified series circuit model, we calculated the *J-V* curve of pn-DSCs through that of the corresponding n-DSC and p-DSC. Pn-DSCs with the same photocathode and different performance photoanode are discussed in this article. The *J*_sc_ of the pn-DSC are determined by the n-DSC, whereas the *V*_oc_ is additive. And the FF of pn-DSC increases when the *J*_sc_ of the n-DSC decrease. Pn-DSCs with a well matched photocurrent show a high *FF* around 65% and achieve an enhanced PCE as compared to the corresponding n-DSC and p-DSC. The results from the calculation show a good agreement with the experimental data. Through adjusting the performance of the photoanode, we got the relatively high *PCE* of 1.33% in pn-DSC which was higher than the corresponding n-DSCs, and an *FF* of 65.3% which could represent good current matching.

## Figures and Tables

**Table 1:** Photovoltaic parameters of the calculated pn-DSCs as well as p-DSCs and n-DSCs.

Condition		*J*_sc_ (mA cm^−2^)	*V*_oc_ (V)	*FF* (%)	*PCE* (%)	*V_max_* (V)
	PSC	2.19	0.15	34.6	0.12	0.09
1	n-DSC	5.26	0.76	67.2	2.68	0.56
	pn-DSC	5.10	0.91	35.2	1.64	0.58
2	n-DSC	3.04	0.79	72.3	1.73	0.62
	pn-DSC	3.04	0.94	54.5	1.56	0.63
3	n-DSC	2.16	0.79	72.3	1.23	0.63
	pn-DSC	2.15	0.94	64.0	1.29	0.68
4	n-DSC	1.01	0.78	73.0	0.57	0.64
	pn-DSC	1.01	0.93	72.1	0.67	0.74

Taken under AM 1.5 solar simulator of 100 mW cm^−2^. Active area = 0.25 cm^2^.

**Table 2:** Photovoltaic parameters of the experimental pn-DSCs as well as p-DSCs and n-DSCs.

	*J*_sc_ (mA cm^2^)	*V*_oc_ (mV)	*FF* (%)	*PCE* (%)
PSC	3.00	98	33.5	0.10
n-DSC-1	3.82	773	68.8	2.03
pn-DSC-1	3.73	855	44.5	1.42
n-DSC-2	2.78	771	72.0	1.54
pn-DSC-2	2.70	895	59.0	1.43
n-DSC-3	2.24	797	73.5	1.31
pn-DSC-3	2.27	899	65.3	1.33
n-DSC-4	1.80	802	73.8	1.06
pn-DSC-4	1.84	912	68.9	1.15

Taken under AM 1.5 solar simulator of 100 mW cm^−2^. Active area = 0.25 cm^2^.

## References

[1] O’Regan B., Grätzel M., Gr M. (1991). A low-cost, high-efficiency solar cell based on dye-sensitized colloidal TiO_2_ films. Nature.

[2] Mathew S., Yella A., Gao P., Humphry-Baker R., Curchod B.F.E., Astani N.A., Tavernelli I., Rothlisberger U., Nazeeruddin K., Grätzel M. (2014). Dye-sensitized solar cells with 13% efficiency achieved through the molecular engineering of porphyrin sensitizers. Nat. Chem..

[3] Yamaguchi T., Uchida Y., Agatsuma S., Arakawa H. (2009). Series-connected tandem dye-sensitized solar cell for improving efficiency to more than 10%. Sol. Energy Mater. Sol. Cells.

[4] Murayama M., Mori T. (2007). Dye-sensitized solar cell using novel tandem cell structure. J. Phys. D Appl. Phys..

[5] Hagfeldt A., Boschloo G., Sun L., Kloo L., Pettersson H. (2010). Dye-Sensitized Solar Cells. Chem. Rev..

[6] He J., Lindström H., Hagfeldt A., Lindquist S.-E. (2000). Dye-sensitized nanostructured tandem cell-first demonstrated cell with a dye-sensitized photocathode. Sol. Energy Mater. Sol. Cells.

[7] Bruder I., Karlsson M., Eickemeyer F., Hwang J., Erk P., Hagfeldt A., Weis J., Pschirer N. (2009). Efficient organic tandem cell combining a solid state dye-sensitized and a vacuum deposited bulk heterojunction solar cell. Sol. Energy Mater. Sol. Cells.

[8] Zhang S., Yang X., Numata Y., Han L. (2013). Highly efficient dye-sensitized solar cells: Progress and future challenges. Energy Environ. Sci..

[9] Nattestad A., Mozer A.J., Fischer M.K.R., Cheng Y.-B., Mishra A., Bäuerle P., Bach U. (2009). Highly efficient photocathodes for dye-sensitized tandem solar cells. Nat. Mater..

[10] Kashif M.K., Nippe M., Duffy N.W., Forsyth C.M., Chang C.J., Long J.R., Spiccia L., Bach U. (2013). Stable Dye-Sensitized Solar Cell Electrolytes Based on Cobalt(II)/(III) Complexes of a Hexadentate Pyridyl Ligand. Angew. Chem. Int. Ed..

[11] Ji Z., Natu G., Huang Z., Wu Y. (2011). Linker effect in organic donor–acceptor dyes for p-type NiO dye sensitized solar cells. Energy Environ. Sci..

[12] Bonomo M., Saccone D., Magistris C., Di Carlo A., Barolo C., Dini D. (2017). Effect of Alkyl Chain Length on the Sensitizing Action of Substituted Non-Symmetric Squaraines for p-Type Dye-Sensitized Solar Cells. ChemElectroChem.

[13] Wood C., Summers G.H., Clark C.A., Kaeffer N., Braeutigam M., Carbone L.R., D’Amario L., Fan K., Farré Y., Narbey S. (2016). A comprehensive comparison of dye-sensitized NiO photocathodes for solar energy conversion. Phys. Chem. Chem. Phys..

[14] Odobel F., Pellegrin Y. (2013). Recent Advances in the Sensitization of Wide-Band-Gap Nanostructured p-Type Semiconductors. Photovoltaic and Photocatalytic Applications. J. Phys. Chem. Lett..

[15] Shalom M., Hod I., Tachan Z., Buhbut S., Tirosh S., Zaban A. (2011). Quantum dot based anode and cathode for high voltage tandem photo -electrochemical solar cell. Energy Environ. Sci..

[16] Barceló I., Guillén E., Lana-Villarreal T., Gómez R. (2013). Preparation and Characterization of Nickel Oxide Photocathodes Sensitized with Colloidal Cadmium Selenide Quantum Dots. J. Phys. Chem. C.

[17] Bonomo M., Di Girolamo D., Piccinni M., Dowling D., Dini D. (2020). Electrochemically Deposited NiO Films as a Blocking Layer in p-Type Dye-Sensitized Solar Cells with an Impressive 45% Fill Factor. Nanomaterials.

[18] Brisse R., Faddoul R., Bourgeteau T., Tondelier D., Leroy J., Campidelli S., Berthelot T., Geffroy B., Jousselme B. (2017). Inkjet Printing NiO-Based p-Type Dye-Sensitized Solar Cells. ACS Appl. Mater. Interfaces.

[19] Nattestad A., Zhang X., Bach U., Cheng Y.-B. (2011). Dye-sensitized CuAlO2 photocathodes for tandem solar cell applications. J. Photon- Energy.

[20] Nakasa A., Usami H., Sumikura S., Hasegawa S., Koyama T., Suzuki E. (2005). A High Voltage Dye-sensitized Solar Cell using a Nanoporous NiO Photocathode. Chem. Lett..

[21] Gibson E.A., Smeigh A., Le Pleux L., Fortage J., Boschloo G., Blart E., Pellegrin Y., Odobel F., Hagfeldt A., Hammarström L. (2009). A p-Type NiO-Based Dye-Sensitized Solar Cell with an Open-Circuit Voltage of 0.35 V. Angew. Chem. Int. Ed..

[22] Qian J., Jiang K., Huang J., Liu Q., Yang L., Song Y. (2012). A Selenium-Based Cathode for a High-Voltage Tandem Photoelectrochemical Solar Cell. Angew. Chem. Int. Ed..

[23] Ho P., Thogiti S., Bao L.Q., Cheruku R., Ahn K.-S., Kim J.H. (2018). Enhanced efficiency via blocking layers at photocathode interfaces in cobalt-mediated tandem dye-sensitized solar cells. Sol. Energy.

[24] Yu M., Natu G., Ji Z., Wu Y. (2012). p-Type Dye-Sensitized Solar Cells Based on Delafossite CuGaO2 Nanoplates with Saturation Photovoltages Exceeding 460 mV. J. Phys. Chem. Lett..

[25] Xiong D., Xu Z., Zeng X., Zhang W., Chen W., Xu X., Wang M.-K., Cheng Y.-B. (2012). Hydrothermal synthesis of ultrasmall CuCrO2 nanocrystal alternatives to NiO nanoparticles in efficient p-type dye-sensitized solar cells. J. Mater. Chem..

[26] Kim J.Y., Kim S.H., Lee H.-H., Lee K., Ma W., Gong X., Heeger A.J. (2006). New Architecture for High-Efficiency Polymer Photovoltaic Cells Using Solution-Based Titanium Oxide as an Optical Spacer. Adv. Mater..

[27] Nithyanandam K., Pitchumani R. (2012). Analysis and design of dye-sensitized solar cell. Sol. Energy.

[28] Koide N., Islam A., Chiba Y., Han L. (2006). Improvement of efficiency of dye-sensitized solar cells based on analysis of equivalent circuit. J. Photochem. Photobiol. A: Chem..

[29] Huang Z., Natu G., Ji Z., He M., Yu M., Wu Y. (2012). Probing the Low Fill Factor of NiO p-Type Dye-Sensitized Solar Cells. J. Phys. Chem. C.

[30] Zhang Y.-D., Huang X.-M., Li D.-M., Luo Y.-H., Meng Q.-B. (2012). How to improve the performance of dye-sensitized solar cell modules by light collection. Sol. Energy Mater. Sol. Cells.

[31] Hanmin T., Xiaobo Z., Shikui Y., Xiangyan W., Zhipeng T., Bin L., Ying W., Tao Y., Zhigang Z. (2009). An improved method to estimate the equivalent circuit parameters in DSSCs. Sol. Energy.

[32] Han L., Koide N., Chiba Y., Mitate T. (2004). Modeling of an equivalent circuit for dye-sensitized solar cells. Appl. Phys. Lett..

[33] Huang Z., Natu G., Ji Z., Hasin P., Wu Y. (2011). p-Type Dye-Sensitized NiO Solar Cells: A Study by Electrochemical Impedance Spectroscopy. J. Phys. Chem. C.

